# Salivary IL-8 as a putative predictive biomarker of radiotherapy response in head and neck cancer patients

**DOI:** 10.1007/s00784-021-04017-0

**Published:** 2021-07-12

**Authors:** Sara Principe, Enrique Zapater-Latorre, Leo Arribas, Enrique Garcia-Miragall, Jose Bagan

**Affiliations:** 1 Faculty of Medicine and Dentistry, University of Valencia, Fundación Investigación Hospital General Universitari (FiHgU) de Valencia, Valencia, Spain; 2 University of Valencia, Hospital General Universitari de Valencia, Valencia, Spain; 3 Department of Radiation Oncology, Fundación Instituto Valenciano de Oncologia, Valencia, Spain; 4 Service of Radiation Oncology, Hospital General Universitari de Valencia, Valencia, Spain; 5 Oral Medicine, Stomatology and Maxillofacial Surgery, University of Valencia, CIBERONC, PI19/00790 Fondo de Investigacion Sanitaria, ISCIII, Hospital General Universitari de Valencia, Avda Tres Cruces, 2, 46014 Valencia, Spain

**Keywords:** IL-8, Saliva, Cytokines, Head and neck cancer, Radiotherapy

## Abstract

**Objectives:**

Ionizing radiation increases the expression of a number of salivary proteins involved in immunoregulatory networks related to infection, injury, inflammation, and cancer. Our main objective was to analyze whether there are significant differences in salivary cytokines before and after radiotherapy and whether any of them are associated to better outcomes after radiotherapy serving as a potential predictive biomarker of response to the treatment.

**Materials and methods:**

We analyzed a panel of eight salivary markers (IL-4, IL-6, IL-8, and IL-10; MCP-1; TNF-α; VEGF; and EGF) in a group of HNC patients (N = 30), before and after irradiation treatment pre- and post-RT. We also compared these results with a group of healthy controls (N = 37). In both groups, we used stimulated saliva and we performed immunoassays based on multi-analyte profiling technology (Luminex xMAP).

**Results:**

In our group of 30 HNC patients, 24 of them showed a good clinical response after radiotherapy treatment while 6 cases did not respond to radiotherapy. The data revealed a post-treatment increase in multiple cytokines in the stimulated saliva of HNC patients; the increases in IL-8 and MCP-1 were statistically significant (p ≤ 0.001 and p ≤ 0.0001, respectively). Analysis of receiver operating characteristic curves indicated the strong potential of IL-8 as a predictive biomarker of RT good outcomes (area under the curve = 0.84; p = 0.018).

**Conclusions:**

After analyzing the panel of salivary cytokines, IL-8 showed the best association to the response to radiotherapy; in this sense, low IL-8 levels in the saliva of HNC patients before receiving irradiation therapy are associated with positive RT outcomes.

**Clinical relevance:**

Salivary IL-8 expression in HNC patients undergoing RT may serve as a potential predictive biomarker of response to the treatment.

## Introduction

Head and neck cancer (HNC) is a complex and heterogeneous pathology, encompassing a variety of tumors that originate from the pharynx, larynx, paranasal sinuses, nasal cavity, salivary glands, and oral cavity [1]. It is the sixth most common neoplasia worldwide, with an incidence estimated at 650,000 cases and 330,000 deaths per year [2]. The majority of HNCs are epithelial tumors, of which 90% are squamous cell carcinomas (SCCs) with various degrees of differentiation [3]. The risk of developing this pathology increases with age, and the majority of cases occur in people aged 50 years or older [4]. Alcohol and tobacco use are among the most common risk factors for HNC [5]. A high-risk human papillomavirus infection, especially of type 16, has recently been implicated in malignant pathogenesis arising from the oropharynx [1]. Survival and recovery benefit from early diagnosis and appropriate therapy; late diagnosis usually requires surgical intervention, often followed by adjuvant radiotherapy (RT) or chemotherapy. Despite many diagnostic and therapeutic advances, prognosis largely depends on the stage at the time of diagnosis. The 5-year survival rate varies from early (70 − 90%) to advanced metastatic stages (40 − 60%), improving in the presence of human papillomavirus [6].

Independent of their cause and origin, the most prominent features in almost all cancers, including HNC, are uncontrolled growth [7] and inflammation [8]. As mediators of inflammation, cytokines are strongly implicated in tumor pathogenesis [7, 9]; they interact with one another in complex ways that may be additive, synergistic, or antagonistic, or involve the induction of one cytokine by another [10]. There is a complex relationship between pro- and anti-inflammatory cytokines, which are secreted in the tumor microenvironment not only by immune cells but also by tumor cells [11]. Pro-inflammatory cytokines are responsible for the growth and proliferation of immune as well as tumor cells; on the other hand, they are also involved in increasing tumor immune surveillance. By contrast, anti-inflammatory cytokines counteract the proliferative potential of pro-inflammatory cytokines and negatively regulate anti-tumor immune responses [11]. The levels of these molecules are generally maintained within a particular range for a particular duration; if not properly maintained, they can induce tissue damage [12]; depending on their balance, the collective effect can be either pro- or anti-tumorigenic [13]. In healthy individuals, cytokines are either undetectable or present at pg mL^−1^ concentrations in bodily fluids and tissues. Elevated concentrations of cytokines indicate activation of the cytokine pathways associated with inflammation or disease progression [14]. For this reason, these proteins are widely used as biomarkers to understand and predict disease progression and its effects on treatment [15]. Because these biomolecules work in networks, it is very important to be able to measure multiple cytokines in a single sample [10].

Ionizing radiation increases the expression of a number of cytokines that are involved in inflammation and wound healing [16]. Alterations in immune and inflammatory, as well as angiogenetic, responses within the HNC microenvironment play a critical role in tumor aggressiveness and its response to RT and chemotherapy, as well as affect the immune system. In HNC, the tumor and surrounding lymphocytes may induce altered cytokine levels [17], and saliva in an oral cancer environment may directly reflect tumor characteristics [11, 18]. Therefore, our main objective was to analyze whether there are significant differences in salivary cytokines before and after radiotherapy, and whether any of them are associated to better outcomes after radiotherapy. Our null hypothesis was that there is no association between salivary cytokines and the response to radiotherapy.

## Materials and methods

The present prospective observational cohort study with patient follow-up was carried out at Valencia University General Hospital (HGUV) between 2017 and 2020. The study was conducted following the fundamental principles established in the Declaration of Helsinki and with prior approval from the Ethics Committee of the University of Valencia (reference H1480791009194) and the Ethics Committee of University General Hospital of Valencia (HGUV) on 19 April 2017.

### Study participants

The study population consisted of two independent groups: group 1, containing 30 HNC patients, and group 2, containing 37 healthy volunteers as controls. Patients were recruited from the Oral and Maxillofacial Surgery; Ear, Nose, and Throat; and Radiotherapy departments at the General University Hospital of Valencia, and the Radiation Oncology Department, Fundación Instituto Valenciano de Oncologia, both in Valencia, Spain. Control subjects were enrolled at the Dental Clinic of the University of Valencia, Spain. An informed consent form was obtained from each participant. The inclusion criteria for group 1 patients were a histological diagnosis of HNC and treatment with RT for at least 30 days, an age of 40 years or older, and an absence of salivary gland disorders. Group 2 included healthy subjects who were sex- and age-matched to group 1 patients without salivary gland alterations. The clinical parameters recorded for the HNC patients were age, sex, diagnosis, location of the lesion, neck metastasis status, TNM classification, treatment tolerance (development of mucositis) [19], and response to the therapy. That last variable was reported at two time points: at the end of the irradiation process (*T* = 0) and 3 months later (*T* = 1). The evaluation of the clinical response, after the use of ionizing radiation, was based on the following classification:

1. Complete response—disappearance of the tumor lesion following RT
2. Partial response—a decrease in tumor size but persistence of the malignant lesion after the irradiation process
3. No response—no decrease in tumor size since the treatment started or unequivocal progression of existing lesions
4. Recurrence—appearance of one or more new lesions at *T* = 1

### Saliva collection

Whole stimulated saliva was collected from all subjects included in this study. For group 1, the first sample was obtained before starting RT and the second sample was collected at 4 − 8 weeks post-RT, with this timeframe used because of the expected inflammatory sequelae subsequent to the therapy. For group 2, saliva specimens were acquired at the beginning of a dental check-up. Participants had to avoid eating, drinking, smoking, or using oral hygiene products for at least 1 h before the procedure. Salivary flow was stimulated by chewing paraffin for 5 min; under continued stimulation, the saliva accumulated in the mouth was expectorated into a 15-mL tube through a funnel [20]. Afterwards, the collected samples were centrifuged at 3000 rpm and 4 °C for 15 min and frozen at − 80 °C until use.

### Cytokine multiplex assay

We performed an immunoassay based on multi-analyte profiling technology (Luminex xMAP) for protein analysis and biomarker screening to quantify the salivary concentrations of interleukin IL-4, IL-6, IL-8, and IL-10; monocyte chemoattractant protein (MCP)-1; tumor necrosis factor (TNF)-α; vascular endothelial growth factor (VEGF); and epidermal growth factor (EGF). We ran the assay using an immunology multiplex assay panel according to the manufacturer’s protocol (Human Cytokine/Chemokine Magnetic Bead Panel, cat. no. HCYTOMAG-60 K; EMD Millipore Corporation, Billerica, MA, USA). Before performing the assay, saliva specimens were thawed and centrifuged at 1500 rpm and 4 °C for 15 min to obtain a clear supernatant devoid of any particles or debris. We diluted each sample twofold, using the assay buffer included in the kit as a diluent. All reagents were warmed to room temperature before starting the experiment. In brief, 25 µL each of assay buffer, sample, or standard and mixed beads were added to each well of a 96-well plate, which was incubated overnight on an orbital shaker at 4 °C. Samples and standards were plated in duplicate. After washing, 25 µL of detection antibody was added to each well, and the samples were incubated for 1 h at room temperature. Following incubation, 25 µL of streptavidin–phycoerythrin was added to each well, and the samples were incubated on an orbital shaker for 30 min at room temperature. The plate was washed, and 150 µL of sheath fluid was added to each well. The levels of fluorescence produced by each standard, quality control, and sample were detected using a multi-analyte profiling analyzer (Luminex 200™; Luminex Corporation, Austin, TX, USA). We then analyzed the data using Bio-Plex Manager software (Bio-Rad Laboratories, Inc., Hercules, CA, USA).

### Statistical analysis

To compare the levels of salivary inflammatory markers pre- and post-RT, we performed the Wilcoxon matched-pairs test. To compare the levels of these proteins between the control subjects and cancer patients pre-treatment, we used a nonparametric Mann–Whitney U test. Values of *p < 0.05, **p < 0.01, and ***p < 0.001 were considered statistically significant.

We conducted the analyses using GraphPad Prism software (ver. 6.0; GraphPad Software Inc., San Diego, CA, USA). Additionally, we performed a correlation analysis, applying Pearson’s pairwise correlation test using the “cor.test()” function in R software. Finally, to identify putative predictive markers of RT response, we applied the nonparametric Mann–Whitney U test to compare HNC responders against nonresponders, followed by a receiver operating characteristic curve analysis. We also used the Mann–Whitney U test to test the means of proteins between two groups (cancer versus controls).

## Results

### Patient characteristics

The most relevant demographic and clinicopathological characteristics of the study population are summarized in Table 1. The HNC patients’ median age was 60.5 years (standard deviation = 13.1); 66.76% were males and 33.33% were females. The most common tumor site was the oral cavity (36.67%), followed by the larynx (33.33%). A SCC histologic description was observed in 96.76% of the cases. Moreover, 60% of the patients were diagnosed at an advanced stage of disease (T3–T4), and 46.67% also presented with neck metastasis.

**Table 1:** Demographic and clinicopathological characteristics of the subjects included in the study

*Study population_Group 1*			*N*	*%*
HNC patients			30	
Median age			60.5	
*Gender*				
Male			20	66.76
Female			10	33.33
*Tumor sites*				
Oral cavity			11	36.67
Pharynx			8	26.67
* Hypopharynx*			(3)	
* Oropharynx*			(5)	
* Nasopharynx*			(0)	
Larynx			10	33.33
Salivary glands			1	3.33
*Histology*				
SCC			29	96.67
ACC			1	3.33
*TNM classification*				
T1			4	13.33
T2			8	26.67
T3			11	36.67
T4			7	23.33
*Neck metastasis*				
Yes			14	46.67
No			16	53.33
*Treatment*				
Surgery + RT			18	60.00
CRT therapy			10	33.33
RT alone			2	6.67
*Development of mucositis*				
Grade I			2	6.67
Grade II			12	40.00
Grade III			13	43.33
NS			3	10.00
*Response to the treatment*	T = 0	%	T = 1	%
Complete response (CR)	29	96.67	24	80.00
Partial response (PR)	1	3.33	1	3.33
No response (NR)	-	-	5	16.67

*HNC* head and neck cancer; *SCC* squamous cell carcinoma; *ACC* adenoid cystic carcinoma; *NS* not specified; *RT* radiotherapy; *CRT* chemoradiation therapy; *T* = 0, end of RT; *T* = 1, 3 months after RT

The development of oral mucositis after RT was observed in 90% of the cases; in particular, two patients developed mild (grade 1) mucositis, 12 patients presented moderate (grade 2) mucositis, and severe (grade 3) mucositis was observed in 13 patients. No cases of grade 4 mucositis were recorded.

Based on treatment responses recorded at *T* = 0 (end of RT), there was a complete response in 96.67% of the patients, although a small decrease to 80.0% was observed at *T* = 1 (3 months after therapy). The control group included 19 healthy males (51.35%) and 18 healthy females (48.65%) with a median age of 57 years (standard deviation = 10.4).

### Evaluation of salivary inflammatory markers

**Fig. 1:**
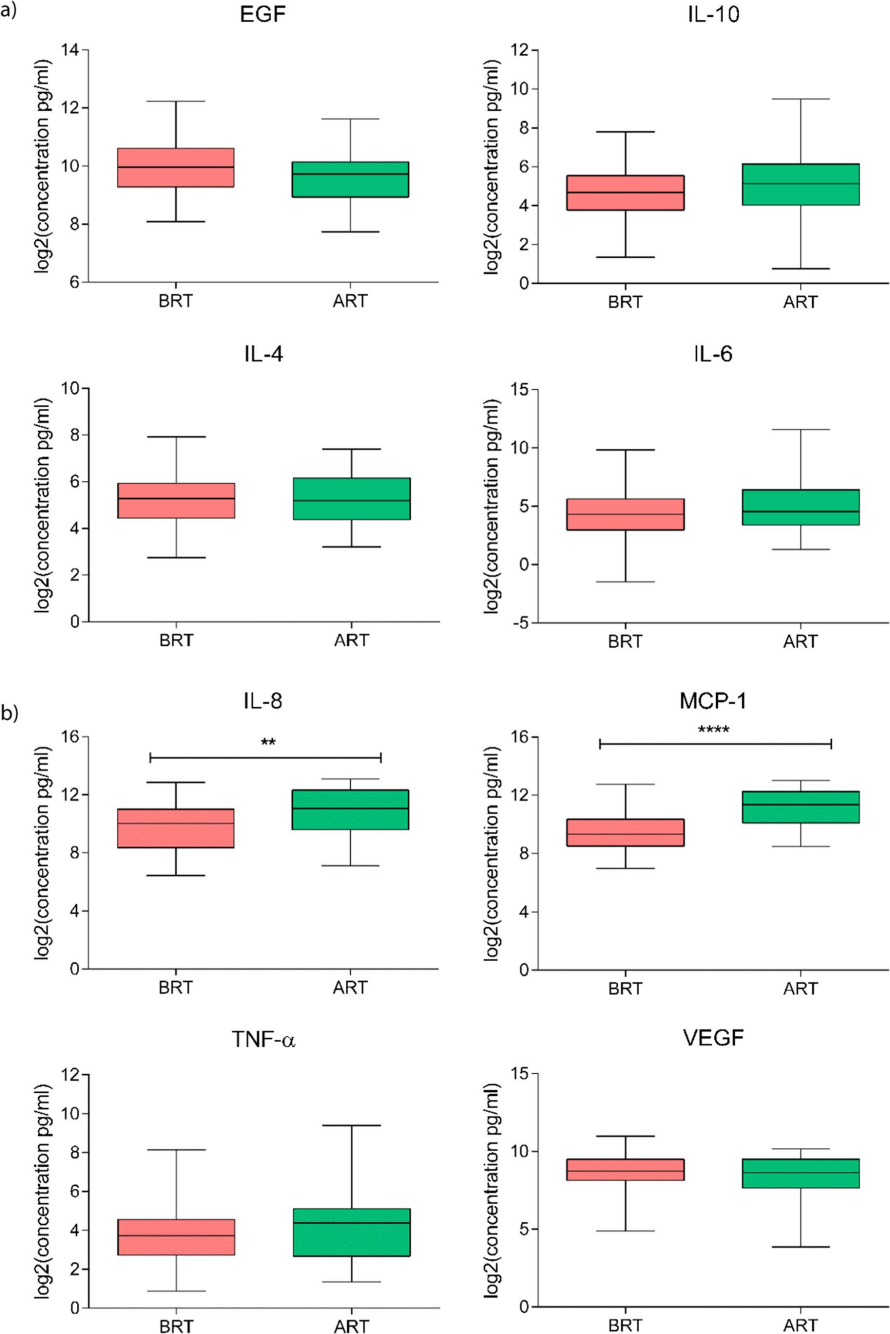
Salivary levels (pg/mL) of EGF; IL-10, IL-4, IL-6, and IL-8; MCP-1; TNF-α; and VEGF detected in head and neck cancer (HNC) patients before (BRT) and after (ART) radiotherapy (*N* = 30). Data are expressed as log_2_(mean signal ratio). Statistical analysis was performed using the Wilcoxon signed-rank nonparametric test, ****p ≤ 0.0001; **p ≤ 0.01

**Fig. 2:**
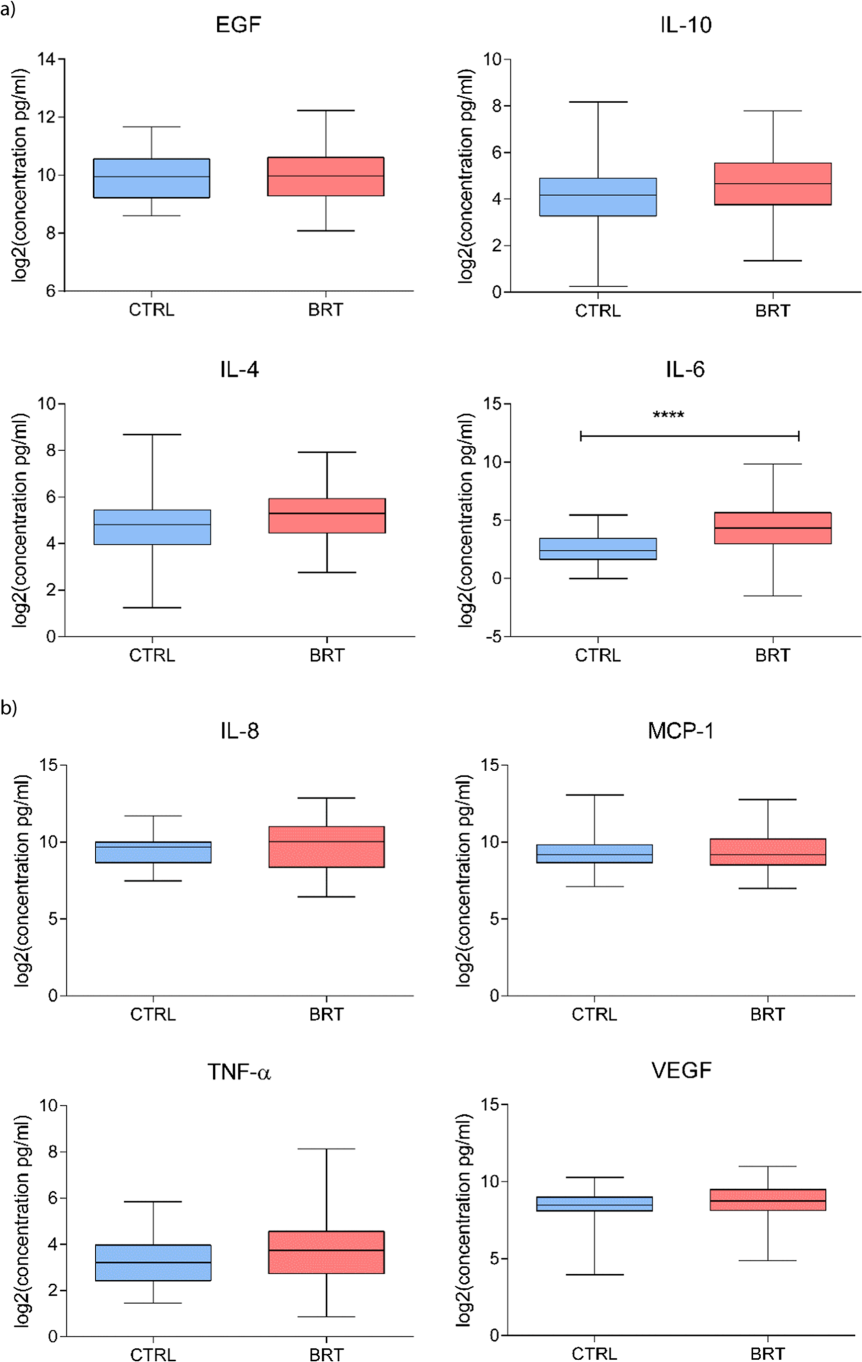
Salivary levels (pg/mL) of EGF, IL-10, IL-4, IL-6, and IL-8, MCP-1, TNF-α, and VEGF detected in control (CTRL) subjects (*N* = 37) and head and neck cancer (HNC) patients before radiotherapy (BRT; *N* = 30). Data are expressed as log_2_(mean signal ratio). Statistical analysis was performed using the nonparametric Mann–Whitney U test; ****p ≤ 0.0001

We first evaluated the salivary levels of pro- (IL-6, IL-8, and TNF-α) and anti-inflammatory cytokines (IL-4 and IL-10), chemokines (MCP-1/CCL2), and growth factors (EGF and VEGF) in HNC patients before and after RT and then compared these levels between control (CTRL) subjects and HNC patients assessed before RT. The data for HNC patients pre- and post-RT revealed that the IL-8 and MCP-1 salivary concentrations increased significantly after RT, with *p* ≤ 0.001 and *p* ≤ 0.0001, respectively. However, no notable changes were observed in the expression levels of IL-10, IL-4, and IL-6; TNF-α; and VEGF. An interesting decrease in EGF levels was detected, albeit without achieving statistical significance (Fig. 1). The salivary protein results showed that various inflammatory markers were higher in the saliva of tumor patients (Fig. 2) with a trend of increased IL-10, IL-4, and IL-8; MCP-1; TNF-α; and VEGF levels observed, probably linked with the disease. However, significance was confirmed only for the concentration of IL-6, with *p* ≤ 0.0001.

Regarding comparison of the salivary cytokines between cancer patients before radiotherapy and the control group, we only found significant differences in the IL-6 levels, which were higher in the cancer group (Mann–Whitney U test = 241; *p* < 0.01). On comparing the cancer patients after radiotherapy versus the controls, we found significantly higher levels in the cancer group for IL-6 (Mann–Whitney U test = 181; *p* < 0.01), IL-8 (Mann–Whitney U test = 268; *p* < 0.01), MCP1 (Mann–Whitney U test = 226; *p* < 0.01), and TNF-α (Mann–Whitney U test = 354; *p* = 0.01).

### Correlation analysis using Pearson’s correlation coefficients

**Fig. 3:**
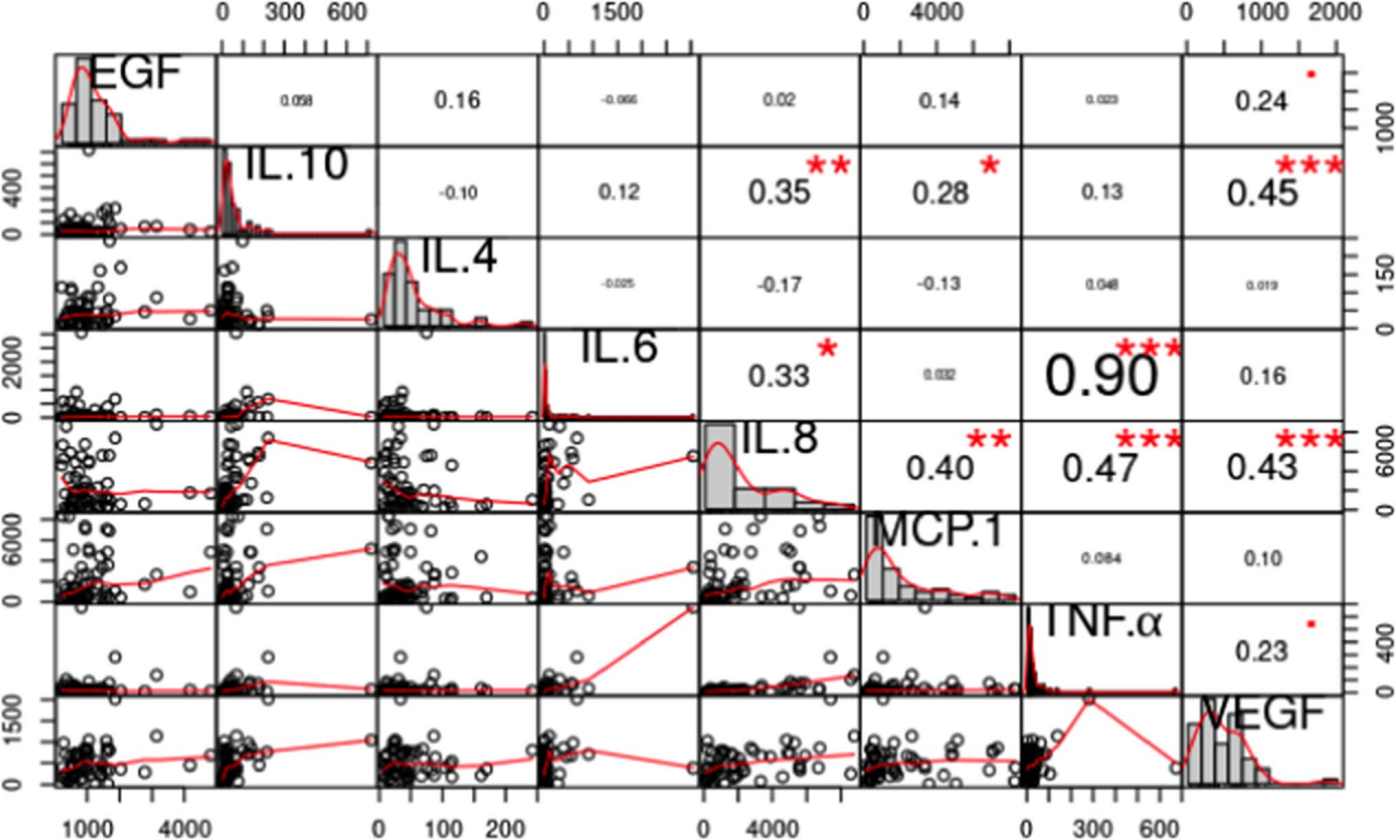
Correlation matrix plot representing patients before (BRT) vs. after (ART) radiotherapy. To be read from the diagonal, where histograms indicate the distributions. The lower panel displays bivariate scatter plots with a fitted line for each possible pairing; the upper panel gives the corresponding Pearson’s correlation coefficient, with text size proportional to its absolute value, and the significance level, each of which is associated with a different *p* value as follows: *** ≤ 0.001, ** ≤ 0.01, * ≤ 0.05

Comparing the HNC patients pre- and post-RT, significant correlations were detected between IL-10 and VEGF, IL-8, and MCP-1; between IL-8 and TNF-α, VEGF, and MCP-1; and between IL-6 and TNF-α and IL-8 (Fig. 3). Thus, patients with an increased IL-8 level also tended to have increased TNF-α, VEGF, and MCP-1 levels.

### Investigation of salivary biomarkers of RT response

The salivary levels of the studied analytes in the HNC patients evaluated before receiving irradiation therapy were compared to the levels post-treatment, to determine whether each concentration was associated with the treatment response recorded at *T* = 1. For this purpose, the HNC patients were divided into two cohorts and classified as responders (*N* = 24), i.e., those that achieved a complete response at the end of the therapy, or nonresponders (*N* = 6), i.e., those that exhibited no or a partial response at *T* = 1. We excluded RT outcomes reported at *T* = 0 from the analysis because the clinical data registered at *T* = 1 were homogeneous.

**Fig. 4:**
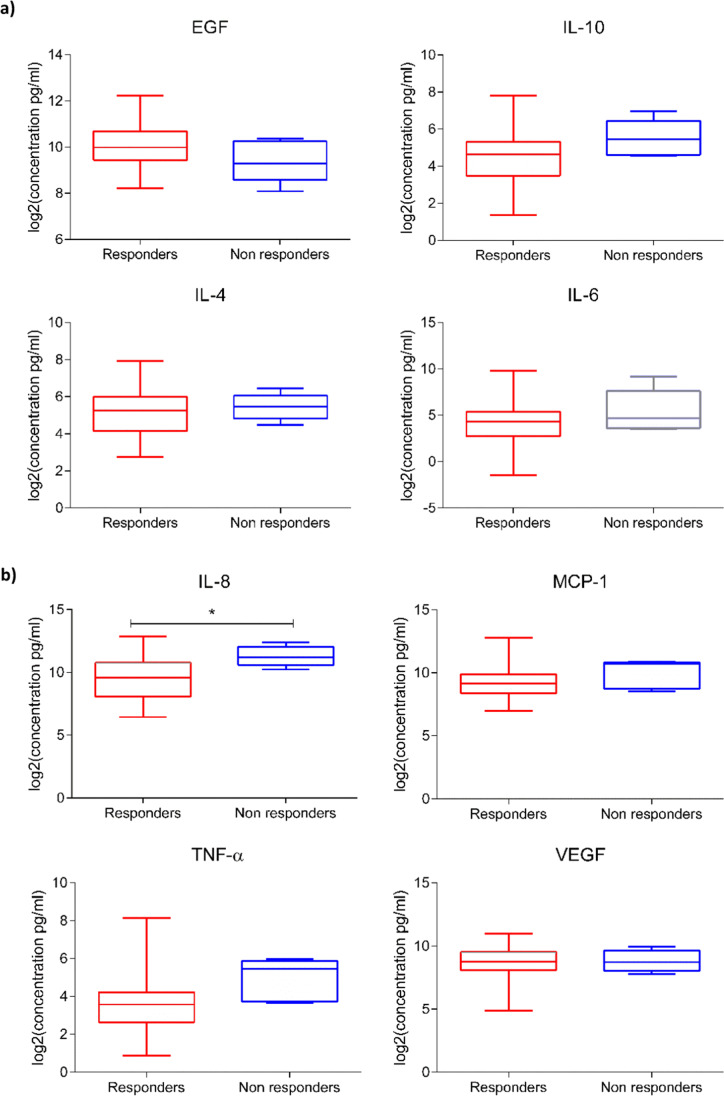
Protein concentration (pg/mL) of EGF; IL-10, IL-4, IL-6, and IL-8; MCP-1; TNF-α; and VEGF detected in the saliva of radiotherapy (RT) responders (*N* = 24) and nonresponders (*N* = 6) in a group of head and neck cancer (HNC) patients evaluated before RT (BRT). Data are expressed as log_2_(mean signal ratio). Statistical analysis was performed using the nonparametric Mann–Whitney U test; *p ≤ 0.05

**Fig. 5:**
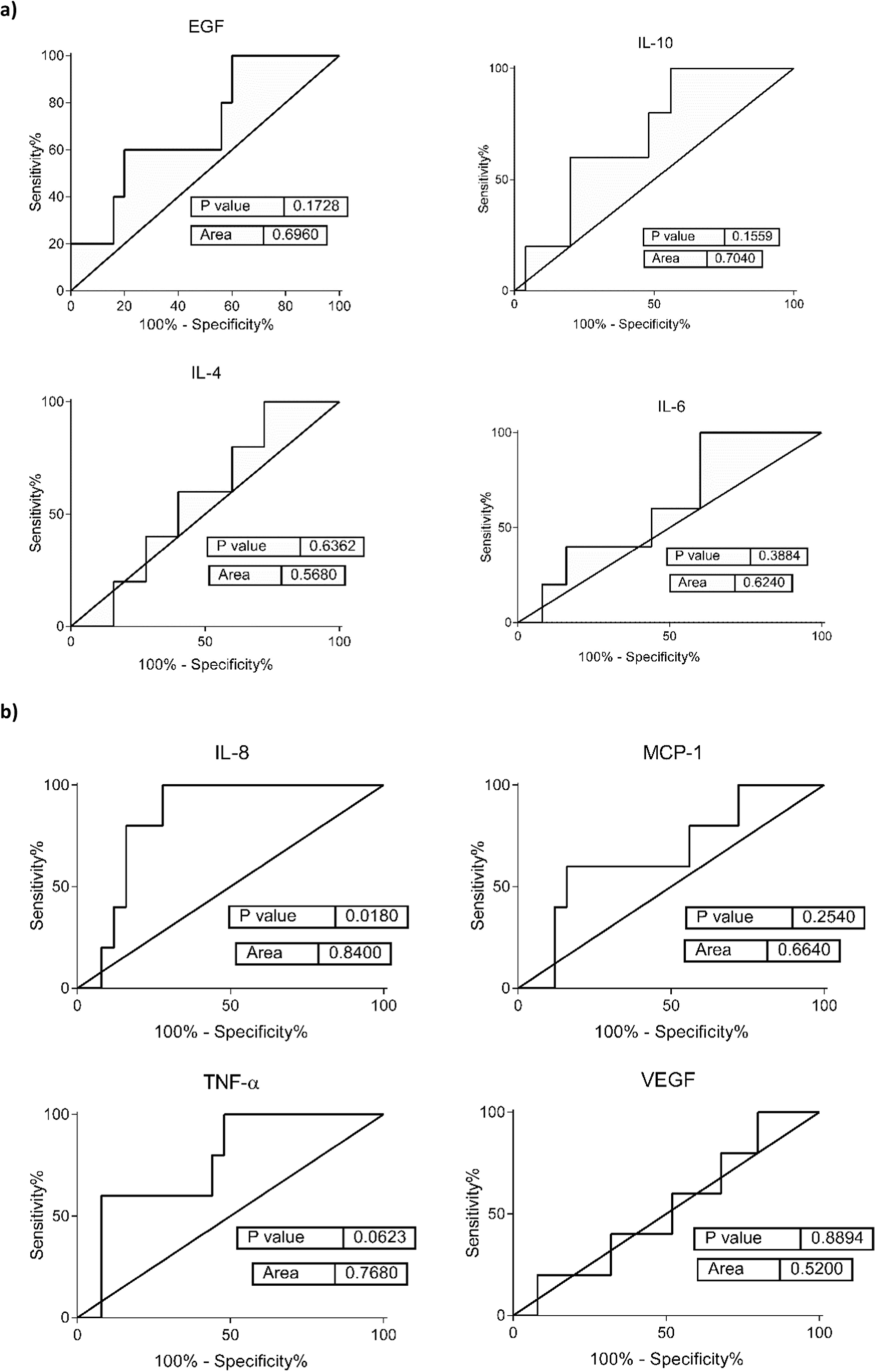
Receiver operating characteristic plots of salivary EGF; IL-10, IL-4, IL-6, and IL-8; MCP-1; TNF-α; and VEGF levels. *P* values and the area under the curve (AUC) are presented. A biomarker with an AUC value > 0.7 in head and neck cancer (HNC) patients evaluated before RT (BRT) treatment was considered informative in discriminating radiotherapy (RT) responders (*N* = 24) from nonresponders (*N* = 6)

The results revealed a trend towards an increase in IL-8, IL-10, IL-4, and IL-6; MCP-1; and TNF-α levels in the saliva specimens of HNC nonresponders, whereas no notable changes were observed in the case of VEGF expression (Fig. 4). However, significance was confirmed only for the protein concentration of IL-8 (*p* ≤ 0.05). Furthermore, the receiver operating characteristic curve analysis substantially confirmed this finding, indicating the strong potential of IL-8 as a predictive biomarker of RT outcomes (area under the curve = 0.84, *p* = 0.018; Fig. 5). A low level of this molecule in the saliva of an HNC patient before receiving RT was associated with a positive treatment response.

## Discussion

Many scientists now emphasize the utility of salivary biomarkers in identifying and managing various diseases. In the field of cancer, markers of inflammation have attracted attention given the role of inflammation in tumorigenesis [21]. The tumor microenvironment consists of cancer, immune, stromal, and inflammatory cells, all of which produce cytokines, growth factors, and adhesion molecules that may promote cancer progression and metastasis [11]. Inflammatory responses and alterations in the immune system play a critical role in disease progression and aggressiveness in HNC, particularly in oral SCC patients [22–24]. The available literature reports that cytokine levels are higher in the saliva of tumor patients, and our data seem to confirm this tendency, with the levels of IL-6, IL-8, TNF-α, IL-4, and IL-10, as well as MCP-1 and VEGF, following a similar trend, i.e., increasing in cancer patients compared to the controls. However, statistical significance was confirmed only for the protein concentration of IL-6. The increase in these inflammatory markers in the saliva of HNC patients before they underwent RT indicates that the markers may be involved in disease progression and severity. However, the majority of salivary investigations for this malignancy [25–28] did not quantify changes pre- to post-treatment, as the present study has done. Among the studied analytes, IL-8 and MCP-1 increased significantly after RT, whereas IL-10, IL-6, and TNF-α were also detected in higher concentrations post-treatment, albeit without achieving statistical significance. Concerning the role of pro-inflammatory cytokines (IL-6, IL-8, and TNF-α), there is evidence that these proteins are produced in a dysregulated manner in oropharyngeal SCC and that they have roles in growth, invasion, the interruption of tumor suppression, immune status, and even survival [29]. IL-6 is a multifunctional cytokine that was originally characterized as a regulator of immune and inflammatory responses [28]; however, under certain conditions, high levels of this molecule may perturb the immune reaction [30–32]. Also, IL-6 can induce the transition from acute to chronic inflammation by recruiting monocytes to the site of inflammation through MCP-1 secretion [33], which may explain the increase also observed for that marker’s level. On the other hand, IL-8 plays an important role in the acute inflammatory response and persists for a relatively long time at the site of inflammation [34]. Besides, the pathological activities of TNF-α are important in early events of tumors, as it regulates a cascade of cytokines, chemokines, adhesions, matrix metalloproteinases, and pro-angiogenic activities [35] and thus may be involved in the mechanism by which inflammation acts as a tumor promoter.

The concurrent increase in IL-6 and IL-8 levels post-treatment suggests a common regulatory mechanism, such as one involving nuclear factor-κB, which plays an important role in the development and progression of head and neck SCC (HNSCC) [36]. The pattern of change evaluated in pro-inflammatory cytokines may provide evidence of acute inflammation after RT [16]. Both MCP-1 and IL-8 induce inflammatory cell recruitment and may reflect local inflammation [16]. The increase in these proteins may be related to the RT response, but other factors to consider are postradiation sequelae, such as mucositis, which is correlated with an increase in cytokine levels [37]. However, in our study, there was no relation between the levels of salivary proteins and the development of mucositis (hence, we did not present our results on this). Furthermore, IL-6 levels have also been found to increase in HNSCC patients who undergo surgery alone [36], suggesting that this increase could be related to an inflammatory response following an invasive procedure and may not be radiation-induced. Nevertheless, RT increases the expression of salivary cytokines, with our results appearing to be consistent with those of other studies [16, 38]. Among the studied proteins, IL-8 was associated with an area under the curve value that was higher than 0.8 and was the only salivary marker whose *p* value was statistically significant. HNC patients with higher levels of IL-8 in pre-treatment samples exhibited a worse response to RT, and vice versa. To our knowledge, the present study is the first to assess the potential role of salivary IL-8 as a predictive biomarker of RT response in HNC.

HNSCC patients may develop recurrent or second primary tumors [39], highlighting the importance of permanently monitoring patients after treatment [36]. Radiation, delivered alone or with chemotherapy, is frequently used in the definitive management of this type of malignancy. Due to concerns for healing during and after the delivery of radiation, the assessment of tumor and normal tissues in the radiated field through biopsies or invasive techniques for correlative assays may be limited. A minimally invasive technique, such as saliva collection, for sampling the local effects of radiation on the tumor and normal tissues may provide a method with which to predict which patients will develop severe toxicity or allow earlier prognosis in the treatment course [16]. Unfortunately, this has been a challenge due to the unavailability of saliva caused by the xerostomia or salivary hypofunction often developed after irradiation treatment [36]. To maximize the likelihood of detecting changes from pre- to post-treatment, we used stimulated saliva. Indeed, a significant advantage of the current study was its longitudinal design, i.e., pre- and post-treatment comparisons of the same patients.

One study limitation was that, regarding the post-treatment saliva samples, we measured cytokine levels within a timeframe of 4 − 8 weeks after irradiation because of the expected inflammatory sequelae subsequent to the therapy; the timeframe was arbitrarily chosen to avoid sequelae, but even so, we cannot be completely confident that all patients had the same health status at the time of sample collection. Given the small sample size, we have to view these as preliminary results that need to be validated in a large cohort of patients. Although saliva exhibits protein changes in response to HNSCC [40] and RT [16, 38], the mechanisms underlying these changes need to be elucidated. Our data do not provide definitive conclusions, but the significant changes reported herein merit further exploration.

### Limitations of the study

This is only a preliminary study evidencing the importance of salivary IL-8 in predicting the possible response to radiotherapy. The limitations of the study are the limited number of cases involved; the fact that the cancers were from different head and neck locations; and finally, the possible influence of chemotherapy treatments associated to some irradiated cancers. All this suggests the need for multicenter studies in which all these limitations are taken into account.

## Conclusions

Monitoring illness status and treatment outcomes through non-invasive means is a desired goal in healthcare. Our results suggest that the sampling of salivary cytokine levels in patients undergoing RT for HNC is feasible and may provide a useful approach with which to identify putative predictive biomarkers in this malignancy. Additional investigations in this field may help with developing a saliva-based test for monitoring pathology status and treatment response, thus improving the prognosis for this disease.
